# Roughing Nitrogen-Doped Carbon Nanosheets for Loading of Monatomic Fe and Electroreduction of CO_2_ to CO

**DOI:** 10.3390/molecules29235561

**Published:** 2024-11-25

**Authors:** Yuxuan Liu, Yufan Tan, Keyi Zhang, Tianqi Guo, Yao Zhu, Ting Cao, Haiyang Lv, Junpeng Zhu, Ze Gao, Su Zhang, Zheng Liu, Juzhe Liu

**Affiliations:** 1 State Key Laboratory of Environmental Criteria and Risk Assessment, Chinese Research Academy of Environmental Sciences, Beijing 100012, China; liuyuxuan@ncepu.edu.cn (Y.L.); zhuyao@craes.org.cn (Y.Z.); caoting@craes.org.cn (T.C.); lvhaiyang@craes.org.cn (H.L.); 2 The Key Laboratory of Resources and Environmental System Optimization, Ministry of Education, College of Environmental Science and Engineering, North China Electric Power University, Beijing 102206, China; 18954531716@163.com (Y.T.); zhangkeyi@ncepu.edu.cn (K.Z.); zhujunpeng@ncepu.edu.cn (J.Z.); gaoze@ncepu.edu.cn (Z.G.); 3 International Institute for Interdisciplinary and Frontiers, Beihang University, Beijing 100191, China; guotianqi@buaa.edu.cn; 4 School of Material Science and Engineering, China University of Petroleum (East China), Qingdao 266580, China; suzhangs@163.com

**Keywords:** single atom, CO_2_ electroreduction, unsaturated coordination, amorphous, rough surface

## Abstract

The catalyst is the pivotal component in CO_2_ electroreduction systems for converting CO_2_ into valuable products. Carbon-based single-atom materials (CSAMs) have emerged as promising catalyst candidates due to their low cost and high atomic utilization efficiency. The rational design of the morphology and microstructure of such materials is desirable but poses a challenge. Here, we employed different Mg(OH)_2_ templates to guide the fabrication of two kinds of amorphous nitrogen-doped carbon nanosheet-supported Fe single atoms (FeSNC) with rough and flat surface structures. In comparison to flat FeSNC with saturated FeN_4_ sites, the rough FeSNC (R-FeSNC) exhibited unsaturated FeN_4−x_ sites and contracted Fe-N bond length. The featured structure endowed R-FeSNC with superior capacity of catalyzing CO_2_ reduction reaction, achieving an exceptional CO selectivity with Faradaic efficiency of 93% at a potential of −0.66 V vs. RHE. This study offers valuable insights into the design of CSAMs and provides a perspective for gaining a deeper understanding of their activity origins.

## 1. Introduction

The electrocatalytic CO_2_ reduction reaction (CRR) presents a promising approach to addressing global energy and environmental challenges by converting CO_2_ to high-valuable chemicals [1,2,3]. Among various products, carbon monoxide (CO) stands out as a pivotal feedstock for the synthesis of multi-carbon chemicals [4,5,6]. Recently, substantial research efforts have been dedicated to developing cost-effective and high-selectivity catalysts for CO_2_-to-CO conversion [7,8,9]. Carbon-based single-atom materials (CSAMs) with distinct structure models have drawn extensive attention for impressive performance and low costs, showing advantages over other common catalysts, e.g., noble metals or molecular materials [10,11,12]. The construction and optimization of such catalysts through morphology control and microstructure modulation represent a promising yet challenging area of exploration.

In terms of morphology control, typical strategies involve the construction of three-dimensional (3D) or two-dimensional (2D) structures, the introduction of pores on the surface, and the augmentation of surface rugosity and curvature [13,14,15,16,17,18]. For instance, the construction of a three-dimensional hierarchical Fe-N-C structure can effectively enhance mass transfer, facilitating the conversion of CO_2_ molecules at single-atom sites [19]. Moreover, the curved carbon surface can provide abundant anchoring sites for single atoms and simultaneously tune their electronic structures, thereby optimizing electrocatalysis reaction energetics [20].

Apart from morphology control, the regulation of microstructures is also of great importance, primarily referring to the regulation on coordination environments of single-atom sites, which encompasses heteroatom doping and defect engineering, etc. [21,22,23,24,25]. The doping of nonmetallic elements (such as O, N, S and P) and the creation of defect sites can effectively modulate the coordination environment and electronic states of metal single atoms, which are closely correlated with their catalytic performance. For example, Zhang et al. designed an asymmetric atomic interface of CuN_3_O/C which could reduce the Gibbs free energy of the CO* desorption step [26]. Zhao et al. created N-defects around Ni sites which can induce a stable pyridinic N dominant Ni-N_2_ unsaturated coordination structure with enhanced kinetics toward the CO_2_-to-CO conversion [27]. In addition, achieving favorable lattice strain through precise control of atomic coordination is also an effective strategy to enhance the electrocatalytic process [28]. For example, the strain regulation of atomically dispersed NiN_4_ active sites in helical carbon could facilitate the generation of *COOH and suppress hydrogen evolution reaction (HER) [29]. On this basis, it is meaningful to develop effective synthetic strategies for modulating the structures of CSAMs and unravel their structure–activity relationships.

Herein, we fabricated flocculent and lamellar Mg(OH)_2_ templates to direct the synthesis of FeSNC catalysts with rough and flat structures, respectively. Rough FeSNC (R-FeSNC) is featured with unsaturated FeN_4−x_ sites and contracted Fe-N bond lengths, which exhibited a considerable CO Faradaic efficiency (FE_CO_) of 93% at −0.66 V vs. RHE and was superior to flat FeSNC (F-FeSNC). Our work provides an efficient strategy to tune the structure and catalytic property of CSAMs.

## 2. Results and Discussion

### 2.1. Material Characterization

**Figure 1:**
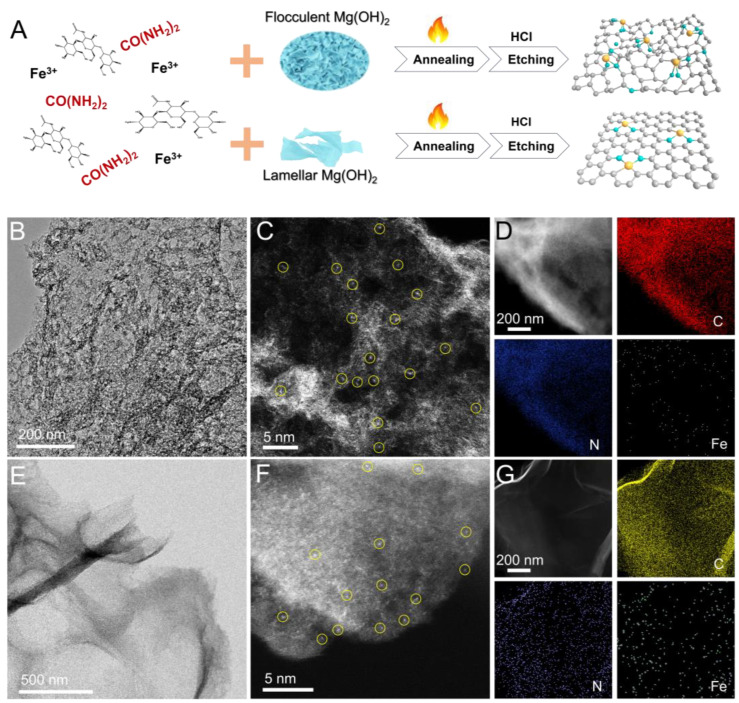
Synthesis and morphology characterization of R-FeSNC and F-FeSNC. (**A**) Schematic illustration of the synthetic route for R-FeSNC and F-FeSNC. (**B**) TEM and (**C**) HAADF-STEM images of R-FeSNC; the yellow circles mark single metal atoms. (**D**) Relevant EDS mapping images. (**E**) TEM and (**F**) HAADF-STEM images of F-FeSNC; the yellow circles mark single metal atoms. (**G**) Corresponding EDS mapping images.

The synthetic scheme is illustrated in Figure 1A. Initially, flocculent and lamellar Mg(OH)_2_ templates are prepared in ethanol and aqueous solutions, respectively (Figures S1 and S2), which are subsequently blended with carboxymethyl chitosan, metal salts (ferrous acetate) and N-source (urea). After freeze-drying and grinding, the mixture underwent calcination. Subsequently, hydrochloric acid etching is employed to remove the templates, resulting in the final products. R-FeSNC and F-FeSNC can be obtained in synthesis procedures involving flocculent and lamellar Mg(OH)_2_ templates, respectively. The X-Ray Diffraction (XRD) patterns in Figure S3 demonstrate the amorphous state of R-FeSNC and F-FeSNC since no sharp Bragg reflection peak is observed. The transmission electron microscopy (TEM) and scanning electron microscope (SEM) images of R-FeSNC show its two-dimensional structure with a rough surface (Figure 1B and Figure S4). Atomically resolved high-angle annular dark-field scanning transmission electron microscopy (HAADF-STEM) imaging reveals the presence of isolated bright dots dispersed on the rough surface of the carbon sheet without metal clusters or particles, which correspond to monatomic Fe sites (Figure 1C). The corresponding energy-dispersive spectroscopy (EDS) elemental mapping images (Figure 1D) demonstrated the uniform distribution of C, N, and Cu on a R-FeSNC nanosheet. In comparison, F-FeSNC exhibits a tawo-dimensional structure with a relatively flat surface (Figure 1E and Figure S5). The relevant HAADF-STEM image also verifies its single-atom distribution state (Figure 1F). The EDS mappings also show homogenous distribution of C, N, and Fe atoms for F-FeSNC in Figure 1G. According to the inductively coupled plasma optical emission spectrometry, the Fe contents of R-FeSNC and F-FeSNC were determined to be 1.3 and 1.0 wt%, respectively. The primary reason for the formation of different morphologies of R-FeSNC and F-FeSNC is attributed to the variation in the morphology of Mg(OH)_2_ templates. Flocculent Mg(OH)_2_, characterized by abundant edge sites and irregular surface undulations, significantly influences the carbon structure formation during the crosslinking and carbonization process when used as a hard template [30,31,32]. This resulted in the final carbon material inheriting some structural characteristics of the template. Consequently, after the template is removed, the R-FeSNC exhibits roughened features. In contrast, the surface of lamellar Mg(OH)_2_ is relatively smooth and even, and the F-FeSNC obtained using it as a template correspondingly exhibits a flat surface.

**Figure 2:**
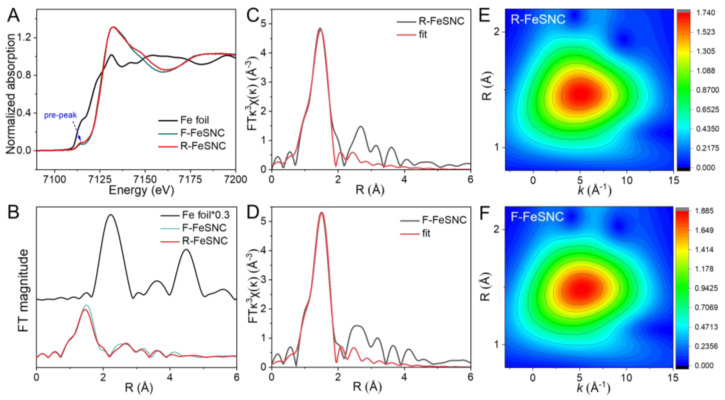
XAS characterization of R-FeSNC and F-FeSNC. (**A**) The normalized Fe K-edge XANES and (**B**) FT-EXAFS spectra of R-FeSNC and F-FeSNC, and the notation "*0.3" appended to "Fe foil" denotes that the peak intensity of the Fe K-edge EXAFS spectrum for the Fe foil has been multiplied by a factor of 0.3. The fitting results of EXAFS spectra for (**C**) R-FeSNC and (**D**) F-FeSNC. The wavelet transforms of (**E**) R-FeSNC and (**F**) F-FeSNC.

The chemical state and coordination of R-FeSNC and F-FeSNC were investigated by X-ray absorption spectroscopy (XAS). The Fe K-edge XANES spectra of R-FeSNC and F-FeSNC exhibit nearly identical absorption edge positions, suggesting that the oxidation states of Fe in both catalysts are approximate. R-FeSNC shows a more dominated pre-peak at 7114 eV than that of F-FeSNC, indicative of its worse coordination symmetry. The Fe K-edge Fourier-transformed extended X-ray absorption fine structure (FT-EXAFS) spectra of R-FeSNC and F-FeSNC exhibit the characteristic peaks of the Fe-N bond at about 1.4 Å, and no Fe-Fe bond (2.1 Å) can be found [33,34]. This result further corroborates the single-atom dispersion of Fe, which is consistent with the HAADF-STEM results. However, the Fe-N peak of R-FeSNC is lower and left-shifted in comparison with F-FeSNC, suggesting their differential coordination environment. This fact can also be proven by EXAFS fitting and wavelet-transform contour plots (Figure 2C–F and Figure S6 and Table S1). The Fe-N coordination numbers for R-FeSNC and F-FeSNC were determined to be approximately 3.5 and 4, respectively, while relevant mean bond lengths were estimated to be 1.99 Å and 2.01 Å, respectively. Apparently, R-FeSNC exhibits an unsaturated coordination configuration of FeN_4−x_ and shorter Fe-N bond length.

**Figure 3:**
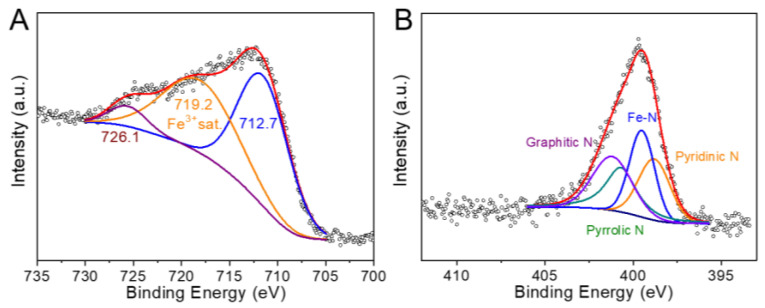
XPS studies of R-FeSNC. (**A**) Fe 2p and (**B**) N1s XPS spectra of R-FeSNC.

The electron and coordination state of R-FeSNC was further studied by X-ray photoelectron spectroscopy (XPS). The peaks at 712.7 eV, 726.1 eV and 719.2 eV are attributed to Fe^3+^, suggesting that the supported Fe single atoms are trivalent [35]. To identify the type of nitrogen, peak decomposition analysis was conducted on N1s (Figure 3B), revealing peaks at 398.8 eV, 399.6 eV, 400.6 eV, and 401.2 eV corresponding to pyridinic nitrogen, Fe-N bonds, pyrrolic nitrogen, and graphitic nitrogen, respectively [36].

### 2.2. Electrocatalytic Performance

**Figure 4:**
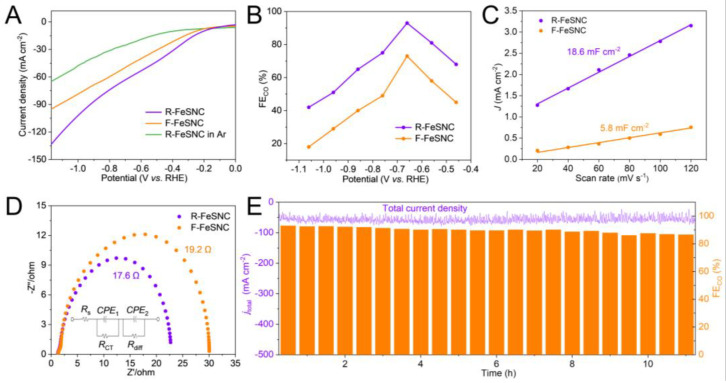
Electrocatalytic performance. (**A**) LSV curves of R-FeSNC and F-FeSNC with CO_2_ and Ar feeding. (**B**) The CO FEs of R-FeSNC and F-FeSNC at different potentials. (**C**) C_dl_ fitting curves. (**D**) EIS data and equivalent circuit diagram fitting. (**E**) Stability test of R-FeSNC at a potential of −0.66 V vs. RHE.

The CRR performance tests of R-FeSNC and F-FeSNC were carried out in a flow cell. The CRR electrocatalytic activity was confirmed by performing linear sweep voltammetry (LSV), feeding CO_2_ or Ar. The larger current density of R-FeSNC with CO_2_ feeding suggests its intrinsic catalytic capacity. The linear sweep voltammetry (LSV) result in Figure 4A demonstrates that the R-FeSNC electrode exhibited a higher total current density in the potential range from −0.46 V to −1.06 V, vs. RHE compared with F-FeSNC, suggesting its superior electrocatalytic activity.

The selectivity of two electrocatalysts was evaluated using FE analysis. It is found that the FE_CO_ of R-FeSNC and F-FeSNC is potential-dependent and exhibited a volcano trend (Figure 4B). R-FeSNC achieved an optimal FE_CO_ of 93% at −0.66 V vs. RHE with a total current density of 60 mA cm^−2^, which is apparently advantageous over F-FeSNC with FE_CO_ of 66% and current density of 45 mA cm^−2^ at same potential. Accordingly, the partial CO current density of R-FeSNC is calculated to be 56 mA cm^−2^, which is nearly double that of F-FeSNC~29 mA cm^−2^. This result demonstrated the superior selectivity and activity of R-FeSNC for CRR. The CO_2_RR performance of R-FeSNC is comparable to state-of-the-art electrocatalysts (Table S2).

The electrocatalytic active surface areas (ECSAs) of two catalysts were evaluated by assessing their double-layer capacitances, which were represented by the linear slopes obtained from plotting the differences in charging current densities against the scan rates (Figure 4C and Figure S7). R-FeSNC demonstrates a linear slope value of 18.6 mF cm^−2^ 3-times higher than that of F-FeSNC (5.8 mF cm^−2^). These strongly suggest that the rough surface endows R-FeSNC with a larger ECSA and more accessible active sites than F-FeSNC. Brunauere–Emmette–Teller (BET) adsorption and desorption curves and specific surface areas of R-FeSNC and F-FeSNC are provided in Figure S8. Accordingly, the specific surface area of R-FeSNC is 1120.846 m^2^/g, which is larger than that of F-FeSNC (873.867 m^2^/g). Electrochemical impedance spectroscopy (EIS) and relevant equivalent circuit diagram fittings were executed to investigate the charge transfer kinetics of two catalysts for CRR. As shown in Figure 4D, R-FeSNC exhibits a smaller circle radius and lower charge transfer resistance (*R*_ct_) of 17.6 Ω than F-FeSNC (19.2 Ω) [37,38]. The electrocatalytic stability of R-FeSNC was determined by chronoamperometric measurements under a constant potential of −0.66 V vs. RHE as displayed in Figure 4E. R-FeSNC demonstrated good stability over 11 h electrolysis with almost unchanged total current density and slightly decreased FE_CO_, which remained above 85%.

**Figure 5:**
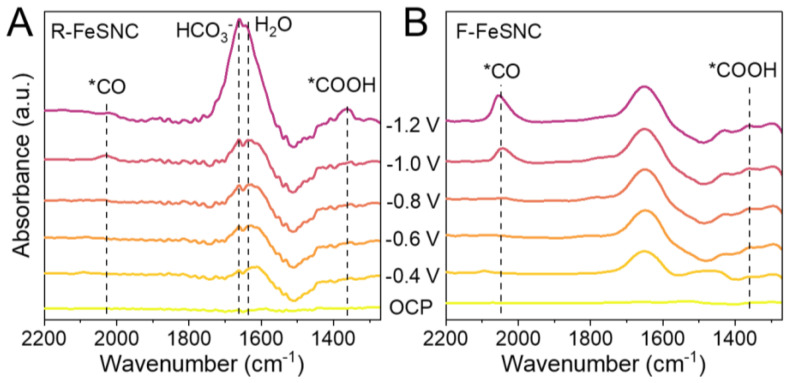
In situ ATR-FTIR spectra of (**A**) R-FeSNC and (**B**) F-FeSNC recorded at different applied potentials for CRR in a CO_2_-saturated 0.5 M KHCO_3_ solution. *CO and *COOH are adsorbed intermediates in the carbon dioxide reduction process.

In order to explore the mechanism, the reaction intermediates generated over the two catalysts during CRR were monitored by in situ electrochemical attenuated total reflectance Fourier-transform infrared (ATR-FTIR) spectroscopy (Figure 5A,B). The peaks at ~2040 and ~1370 cm^−1^ can be assigned to linear-adsorbed *CO and *COOH species, respectively, which are key intermediates for electrochemical CO_2_-to-CO transformation [39,40]. As applied potential increased, a more intense *COOH peak was observed for R-FeSNC than F-FeSNC, suggesting its facilitation of the process of CO_2_ (g) + H^+^ + e^−^ → *COOH. After that, *COOH would cause protons and electrons to form *CO (*COOH + H^+^ + e^−^ → *CO + H_2_O) followed by *CO desorption (*CO → CO (g)). In addition, R-FeSNC exhibited less distinct *CO peaks than F-FeSNC, demonstrating that CO was easily desorbed from its surface. Meanwhile, R-FeSNC showed more obvious and differentiated peaks at 1660 and 1635 cm^−1^ which originate from the stretching of absorbed H_2_O and HCO_3_^−^ molecules [41]. The existence of H_2_O and HCO_3_^−^ molecules could contribute to the formation of H^+^ via the dissociation processes (HCO_3_^−^ → H^+^ + CO_3_^2−^ and H_2_O → H^+^ + OH^−^), thereby facilitating the formation of *COOH and *CO. Thus, the CO_2_-to-CO electroreduction process should be more favorable on R-FeSNC than F-FeSNC.

As aforementioned, R-FeSNC exhibits higher activity and selectivity for the electrocatalytic reduction of CO_2_ to CO, which is presumably closely related to its unique structural characteristics. Firstly, its rough surface can prevent the stacking of carbon nanosheets, thereby enhancing the solid–liquid contact area and consequently increasing the ECSA [42,43]. Secondly, the pits or channels on the rough surface can create specific localized environments, which may provide favorable conditions for the adsorption of H_2_O and HCO_3_^-^, as evidenced by in situ ATR-FTIR. Thirdly, the amorphous rough surface, featuring abundant edges and dramatic structural fluctuations, can affect the coordination number and form of anchored Fe single atoms and N, leading to the formation of unsaturated FeN_4−x_ sites and shorter Fe-N bonds [44]. The unsaturated atomic coordination configuration and shortened bond distance can induce polarized charge distributions and an up-shifted total density of states towards the Fermi level which are conductive to stabilizing the key intermediate, i.e., *COOH, and accelerate charge transfers [45,46,47]. The characteristic microstructures should be conducive to optimizing the electronic and spatial structure of the active sites, thereby facilitating the kinetics of multi-step reactions.

## 3. Materials and Methods

### 3.1. Materials

All chemicals and reagents were analytical reagents (ARs). Ethanol (EtOH, C_2_H_5_OH) was obtained from Tianjin Okaibo Chemical Co., Ltd. (Tianjin, China). Potassium hydroxide (KOH) was purchased from Shanghai Macklin Biochemical Technology Co., Ltd. (Shanghai, China). Magnesium nitrate tetrahydrate (≥99.98% metals basis), carboxymethyl chitosan, and iron nitrate 9-hydrate (≥99.9% metals basis) were obtained from Shanghai Aladdin Biochemical Technology Co., Ltd. (Shanghai, China).

### 3.2. Catalyst Preparation

As is typical, 2.2 g of magnesium nitrate tetrahydrate was dissolved into 100 mL of ethanol and stirred for 0.5 h. After that, 1 g of NaOH was dissolved into 10 mL of water and then injected into the above solution immediately. The precipitate was centrifuged, washed, and dried overnight. Finally, flocculent Mg(OH)_2_ was obtained. The lamellar Mg(OH)_2_ was fabricated just by changing ethanol for dissolving magnesium nitrate tetrahydrate into water.

The rough R-FeSNC and F-FeSNC were fabricated for structure and performance comparison. For synthesis of R-FeSNC, 0.5 g of flocculent Mg(OH)_2_ and 6.2 mg of FeCl_3_ were dispersed into 30 mL of water by sonication for 0.5 h. After that, 0.5 g of carboxymethyl chitosan was dissolved into above suspension, which was then freeze-dried. R-FeSNC was synthesized by annealing the above-obtained powder at 900 ℃ for 1 h in an Ar atmosphere and acid pickling to remove templates. F-FeSNC can be synthesized following the above steps, except for changing flocculent Mg(OH)_2_ to lamellar Mg(OH)_2_.

### 3.3. Catalyst Characterization

X-ray diffraction (XRD, Rigaku SmartLab SE, Rigaku, Tokyo, Japan) was used to confirm crystal properties. X-ray photoelectron spectroscopy (XPS) was tested on the thermal ESCALAB 250xi spectrometer (Thermo Fisher, Waltham, MA, USA) and the peak binding energy of C1s 284.8 eV was used for calibration of all peaks. The microstructure of the catalyst was characterized by transmission electron microscopy (TEM, TALOS F200S, Thermo Fisher, Waltham, MA, USA) with an energy dispersive spectrometer (EDS). XAS spectra were obtained from the Beijing Synchrotron Radiation Facility at 1W1B (Beijing Electron–Positron Collider II, China). Fe foil was measured for energy calibration. BET surface area was measured with Quantachrome, Autosorb-iQ. The in situ electrochemical attenuated total reflectance Fourier transform infrared (ATR-FTIR, Shimadzu IRTracer-100, Kyoto, Japan) was carried out to obtain the chemical bonds or functional information of different materials.

### 3.4. Preparation of Gas Diffusion Electrodes (GDEs)

Carbon paper (Sinero YLS-30T) with a microporous layer was purchased from the Sinero Store (Suzhou Sinero Technology, Suzhou, China). 20 mg of catalysts (R-FeSNC and F-FeSNC) were dispersed in 4 mL ethanol with addition of 80 uL of 5 wt% Nafion solution. The ink was placed into the ultrasonic machine for one hour to ensure uniform dispersion. After that, the prepared ink was evenly sprayed on a GDE (3 cm × 8 cm) through the spray gun, and the weight of the sprayed catalyst was determined by the weight difference in GDE before and after airbrushing. The loading of catalyst was controlled at 0.3 mg cm^−2^. The GDEs were cut to 1 cm × 3 cm and dried in a vacuum overnight at 60℃ before using.

## 4. Conclusions

In summary, we manipulated the morphology of amorphous N-doped carbon-supported, isolated Fe atoms using diverse Mg(OH)_2_ templates. Flocculent and lamellar Mg(OH)_2_ templates lead to R-FeSNC and F-FeSNC, respectively. The R-FeSNC features a rough surface and unsaturated FeN_4−x_ sites. In CRR electrocatalysis, R-FeSNC demonstrated satisfactory selectivity and activity with an FE_CO_ of 93% at a current density of 60 mA cm^−2^, superior to F-FeSNC with an FE_CO_ of 73% and a current density of 45 mA cm^−2^. The enhanced performance of R-FeSNC can be attributed to well-designed morphology and microstructure that can increase ECSA, create specific localized environments and optimize active sites. This work paves the way to building highly efficient and selective CO_2_RR catalysts by modulating the morphology and microstructure of CSAMs.

## Data Availability

Data are contained within the article and Supplementary Materials.

## Supplementary Materials

The following supporting information can be downloaded at: https://www.mdpi.com/article/10.3390/molecules29235561/s1. Figure S1: The SEM images of flocculent Mg(OH)_2_. Figure S2: The SEM images of lamellar Mg(OH)_2_. Figure S3: The XRD patterns of R-FeSNC and F-FeSNC. Figure S4: The SEM image of R-FeSNC. Figure S5: The SEM image of F-FeSNC. Figure S6: EXAFS curves-fitting results of Fe K-edge of Fe foil. Figure S7: CV curves of (A) R-FeSNC and (B) F-FeSNC at varied scan rates (20, 40, 60, 80, 100 and 120 mV s-1). Figure S8: BET adsorption and desorption curves and surface area of (A) R-FeSNC and (B) F-FeSNC. Table S1: EXAFS fitting parameters extracted from the Fe K-edge. Table S2: Performance comparison of R-FeSNC with some advanced catalysts. References [7,8,9,48,49,50,51,52,53,54] are cited in the supplementary materials.
