# Supplementary material for: Roughing Nitrogen-Doped Carbon Nanosheets for Loading of Monatomic Fe and Electroreduction of CO2 to CO
Source: Molecules. 2024 Nov 25;29(23):5561. doi: 10.3390/molecules29235561 (PMC11643360; doi:10.3390/molecules29235561)
Supplement: Supplementary file 1 [file molecules-29-05561-s001.zip › molecules-3305642-supplementary.pdf]

Electronic Supplementary Material

for

Roughing Nitrogen-Doped Carbon Nanosheets for Loading of Monatomic Fe and Electroreduction of CO_2 to CO

Yuxuan Liu [1,2], Yufan Tan [2], Keyi Zhang [2], Tianqi Guo [3], Yao Zhu [1], Ting Cao [1], Haiyang Lv [1], Junpeng Zhu [2], Ze Gao [2], Su Zhang [4], Zheng Liu [1,*] and Juzhe Liu [2,*]

1 State Key Laboratory of Environmental Criteria and Risk Assessment, Chinese Research Academy of Environmental Sciences, Beijing 100012, China; liuyuxuan@ncepu.edu.cn (Y.L.); zhuyao@craes.org.cn (Y.Z.); caoting@craes.org.cn (T.C.); lvhaiyang@craes.org.cn (H.L.)

2 The Key Laboratory of Resources and Environmental System Optimization, Ministry of Education, College of Environmental Science and Engineering, North China Electric Power University, Beijing 102206, China; 18954531716@163.com (Y.T.); zhangkeyi@ncepu.edu.cn (K.Z.); zhujunpeng@ncepu.edu.cn (J.Z.); gaoze@ncepu.edu.cn (Z.G.)

3 International Institute for Interdisciplinary and Frontiers, Beihang University, Beijing 100191, China; guotianqi@buaa.edu.cn

4 School of Material Science and Engineering, China University of Petroleum (East China), Qingdao 266580, China; suzhangs@163.com

* Correspondence: liuzheng@craes.org.cn (Z.L.); liujuzhe@ncepu.edu.cn (J.L.)

Contents

Texts

Text S1. Electrochemical measurements.

Figures

Figure S1. The SEM images of flocculent $Mg(OH)_2$.

Figure S2. The SEM images of lamellar $Mg(OH)_2$.

Figure S3. The XRD patterns of R-FeSNC and F-FeSNC.

Figure S4. The SEM image of R-FeSNC.

Figure S5. The SEM image of F-FeSNC.

Figure S6. EXAFS curve—fitting results of Fe K-edge of Fe foil.

Figure S7. CV curves of (A) R-FeSNC and (B) F-FeSNC at varied scan rates (20, 40, 60, 80, 100 and 120 mV s^{-1}).

Figure S8. BET adsorption and desorption curves and surface area of (A) R-FeSNC and (B) F-FeSNC.

Tables

Table S1. EXAFS fitting parameters extracted from the Fe K-edge.

Table S2. Performance comparison of R-FeSNC with some advanced catalysts.

References

Texts

Text S1. Electrochemical measurements

The gas–tight flow cell system comprised a gas compartment and two liquid compartments with channels (2.0 cm × 0.5 cm × 0.3 cm). The CO_2 gas and 1 M KOH electrolyte was introduced into the system at a flow rate of 10 mL min^{-1}. GDEs were used to separate the CO_2 gas and liquid electrolyte. An anion exchange membrane (FAB-PK-130) (Fuel Cell Store) was sandwiched between the two PEEK sheets to separate the chambers. Nickel foam served as the anode material. All potentials were measured against an Ag/AgCl reference electrode saturated with KCl solution and then converted to potentials versus the reversible hydrogen electrode (RHE) using the following equation: E(RHE) = E(Ag/AgCl) + 0.0592 × pH + 0.197.

Electrochemical impedance spectroscopy (EIS) was conducted on the CHI 760E electrochemical workstation at the potential of −1.3 V in the frequency from 0.01 Hz to 100 kHz with a signal amplitude of 5 mV.

The gas phase composition was analyzed by gas chromatograph (GC) equipped with a flame ionization detector (FID) and thermal conductivity detector (TCD) every 15 min. Ultrahigh purity Ar (99.999%) was used as the carrier gas. The volume ratio of gaseous products was calibrated by standard curves from standard gasses. The Faraday efficiency (FE) of the gas product was calculated based on the following equations:

$$FE_{gas}\% = \frac{Q_i}{Q_{total}} \times 100\% = \frac{Z_i \times P_0 \times V_0 \times v(vol\%) \times F}{R \times T \times I \times 60(s\,min^{-1})} \times 100\%$$

Z_i: the electron transfer number for product formation;
P_0: one atmosphere, 1.013 × 10^5 Pa;
V_0: gas flow rate measured by a flow meter;
$v(vol\%)$: volume concentration of certain gas product in the exhaust gas from the cell;
F: Faradaic constant (96,485 C mol^{-1});
R: universal gas constant (8.314 J mol^{-1} K^{-1});
T: the reaction temperature (298 K);
I: the average current during the sample injection.

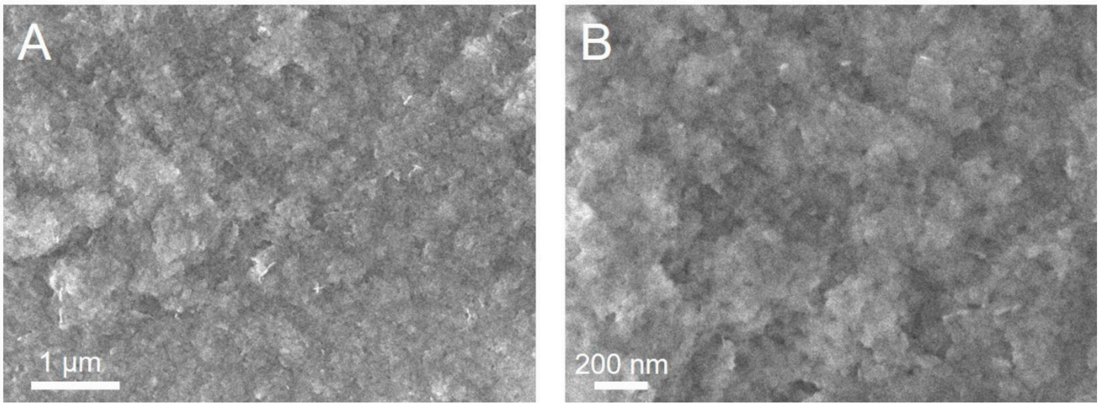

Figure S1. The SEM images of flocculent $Mg(OH)_2$.

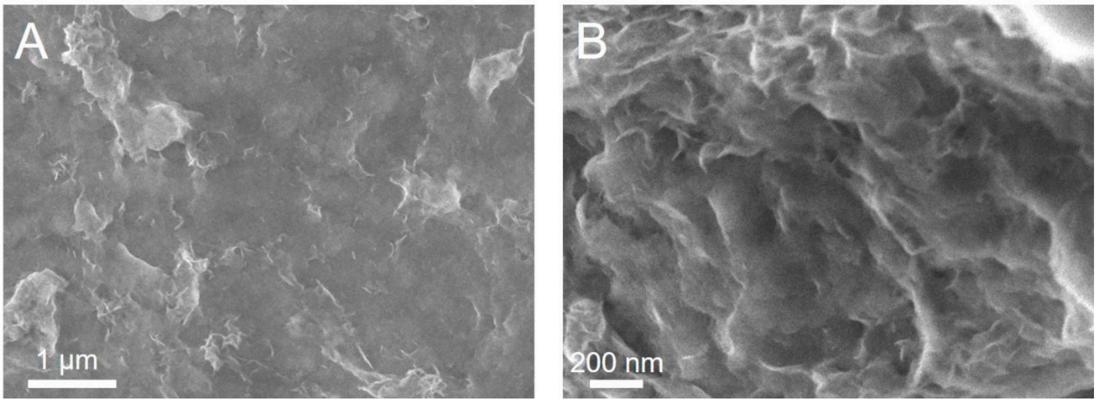

Figure S2. The SEM images of lamellar $Mg(OH)_2$.

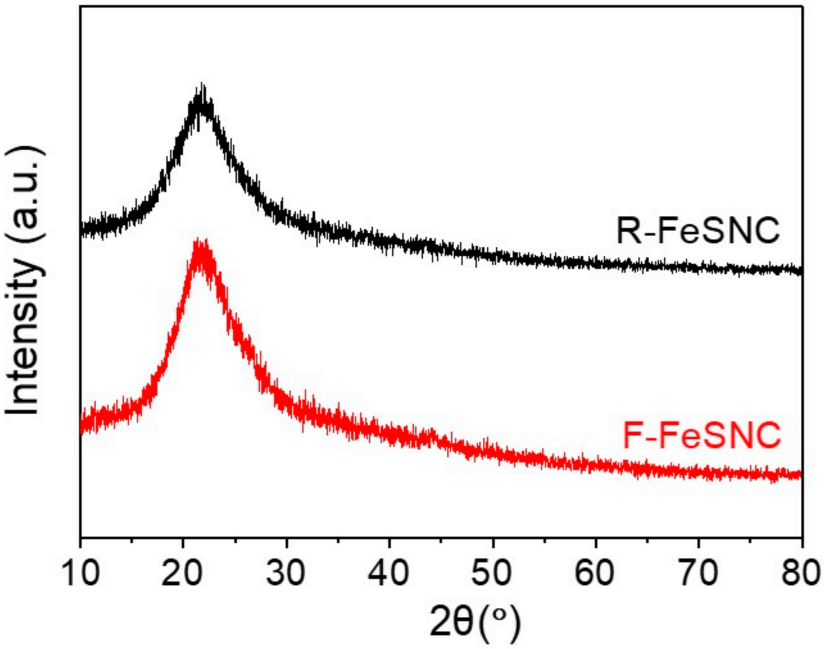

Figure S3. The XRD patterns of R-FeSNC and F-FeSNC.

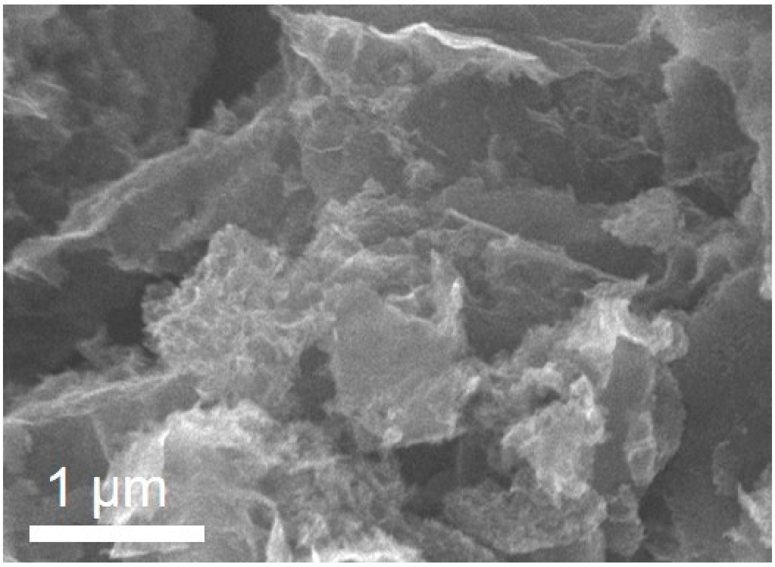

Figure S4. The SEM image of R-FeSNC.

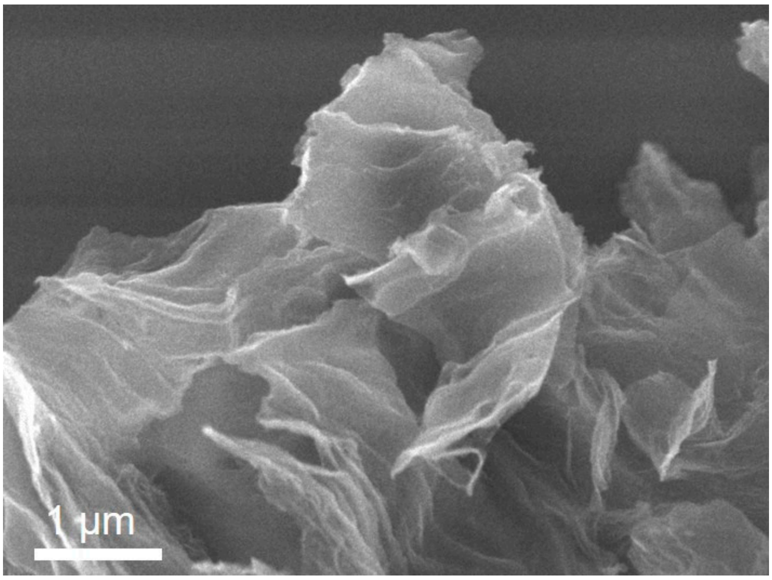

Figure S5. The SEM image of F-FeSNC.

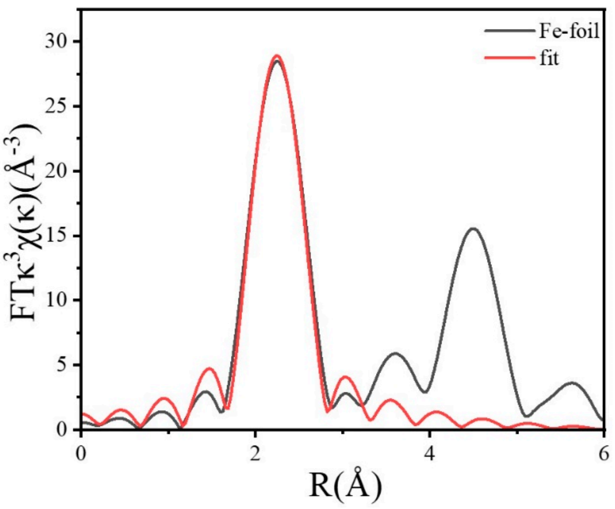

Figure S6. EXAFS curves—fitting results of Fe K-edge of Fe foil.

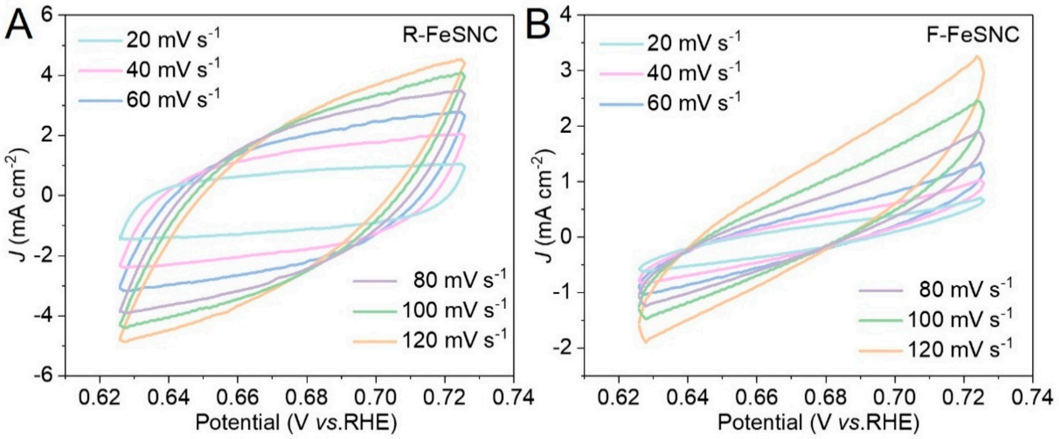

Figure S7. CV curves of (A) R-FeSNC and (B) F-FeSNC at varied scan rates (20, 40, 60, 80, 100 and 120 mV s^{-1}).

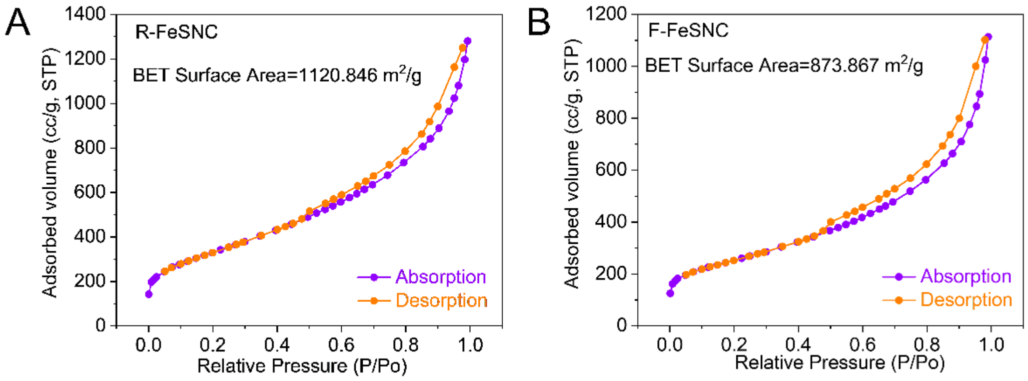

Figure S8. BET adsorption and desorption curves and surface area of (A) R-FeSNC and (B) F-FeSNC.

Table S1. EXAFS fitting parameters extracted from the Fe K-edge (S_0^2=0.78). The obtained XAFS data were processed in Athena (version 0.9.25) for background, pre-edge line and post-edge line calibrations. Then, Fourier-transformed fitting was carried out in Artemis (version 0.9.25). The k^3-weighting, a k-range of 2.7-10.5 Å $^{-1}$ and R-range of 1-1.8 Å were used for the fitting of R-FeSNC and F-FeSNC. The k^3-weighting, k-range of 2.7-12 Å $^{-1}$ and R range of 1-3 Å were used for fitting Fe-foil. Error bounds that characterize the structural parameters obtained by EXAFS spectroscopy were estimated as N ± 20%, R ± 1%, σ^2 ± 20%, ΔE_0 ± 20%. C.N.: coordination numbers; R: bond distance; σ^2: Debye–Waller factors; ΔE: the inner potential correction. R factor: goodness of fit.

Sample	Path	C.N.	R (Å)	σ^2 (Å ^{2})	ΔE_0 (eV)	R factor
R-FeSNC	Fe-N	3.5	1.98	0.010	-5.82	0.0019
F-FeSNC	Fe-N	4	2.01	0.008	-5.15	0.0005
Fe-foil	Fe-Fe	12	2.61	0.004	-2.72	0.01

Table S2. Performance comparison of R-FeSNC with some advanced catalysts.

Catalyst	Electrolyte	FE_{CO} (%)	current density	Reference
R-FeSNC	1 M KOH	93	~60 mA cm^{-2}	This work
[a]Pd concave cubes	0.1 M $KHCO_3$	90.6	~5 mA cm^{-2}	Ref. [1]
[b]OD-Ag	0.1 M $KHCO_3$	89	1.15 mA cm^{-2}	Ref. [2]
[c]PcCu-O_8-Zn	0.1 M $KHCO_3$	88	~4 mA cm^{-2}	Ref. [3]
ZIF-8	1 M KOH	61.1	24.55 mA cm^{-2}	Ref. [4]
Ni-N-Gr	0.1 M $KHCO_3$	~90	~2.5 mA cm^{-2}	Ref. [5]
[d]CALF-20	1 M KOH	94.5	34.48 mA cm^{-2}	Ref. [4]
Cu-0	0.1 M $KHCO_3$	78	13.2 mA cm^{-2}	Ref. [6]
Zn-1.7	0.5 M $KHCO_3$	~50	~1.7 mA cm^{-2}	Ref. [7]
Pd_{20}-Ni_{80}/ZC	1 M KOH	95.3	200 mA cm^{-2}	Ref. [8]
[e]AgEN40	0.5 M $KHCO_3$	83	1.9 mA cm^{-2}	Ref. [9]
CdS	0.5 M KOH	91.3	212 mA cm^{-2}	Ref. [10]

[a]Pd concave cubes: A Pd of concave cubes structure corresponding to cubes and octahedrons.

[b]OD-Ag: An oxide-derived nanostructured Ag catalyst.

[c]PcCu-O8-Zn: A layer-stacked, bimetallic two-dimensional conjugated metal–organic framework (2D c-MOF) with copper-phthalocyanine as ligand (CuN4) and a zinc–bis(dihydroxy) complex (ZnO4).

[d]CALF-20: A Zn-based MOF and azolate functional ligand, which is 1,2,4-triazole (Calgary Framework 20, CALF20).

[e]AgEN40: Ethylenediamine (EN) as an additive, changed the electrodeposition form of silver.

References

1. Dong, H.; Zhang, L.; Yang, P. P.; Chang, X. X.; Zhu, W. J.; Ren, X. H.; Zhao, Z. J.; Gong, J. L., Facet Design Promotes Electroreduction of Carbon Dioxide to Carbon Monoxide on Palladium Nanocrystals. *Chem. Eng. Sci.* **2019,** 194, 29-35.
2. Ma, M.; Trzesniewski, B. J.; Xie, J.; Smith, W. A., Selective and Efficient Reduction of Carbon Dioxide to Carbon Monoxide on Oxide-Derived Nanostructured Silver Electrocatalysts. *Angew. Chem. Int. Edit.* **2016,** 55, (33), 9748-9752.
3. Zhong, H. X.; Ghorbani-Asl, M.; Ly, K. H.; Zhang, J. C.; Ge, J.; Wang, M. C.; Liao, Z. Q.; Makarov, D.; Zschech, E.; Brunner, E.; Weidinger, I. M.; Zhang, J.; Krasheninnikov, A. V.; Kaskel, S.; Dong, R. H.; Feng, X. L., Synergistic Electroreduction of Carbon Dioxide to Carbon Monoxide on Bimetallic Layered Conjugated Metal-organic Frameworks. *Nat. Commun.* **2020,** 11, (1), 10.
4. Al-Attas, T. A.; Marei, N. N.; Yong, X.; Yasri, N. G.; Thangadurai, V.; Shimizu, G.; Siahrostami, S.; Kibria, M. G., Ligand-Engineered Metal-Organic Frameworks for Electrochemical Reduction of Carbon Dioxide to Carbon Monoxide. *ACS Catal.* **2021,** 11, (12), 7350-7357.
5. Su, P.; Iwase, K.; Nakanishi, S.; Hashimoto, K.; Kamiya, K., Nickel-Nitrogen-Modified Graphene: An Efficient Electrocatalyst for the Reduction of Carbon Dioxide to Carbon Monoxide. *Small* **2016,** 12, (44), 6083-6089.
6. Huang, P.; Chen, J. X.; Deng, P. L.; Yang, F.; Pan, J.; Qi, K.; Liu, H. F.; Xia, B. Y., Grain Refinement of Self-Supported Copper Electrode by Multiple-Redox Treatment for Enhanced Carbon Dioxide Electroreduction towards Carbon Monoxide Generation. *J. Catal.* **2020,** 381, 608-614.
7. Kim, J.; Kim, H.; Han, G. H.; Ahn, S. H., Solution-phase-reconstructed Zn-based Nanowire Electrocatalysts for Electrochemical Reduction of Carbon Dioxide to Carbon Monoxide. *Int. J. Energy Res.* **2021,** 45, (5), 7987-7997.
8. Wijaya, D. T.; Haryanto, A.; Lim, H.; Jin, K.; Lee, C., Sub-2 nm Mixed Metal Oxide for Electrochemical Reduction of Carbon Dioxide to Carbon Monoxide. *J. Energy Chem.* **2023,** 84, 303-310.
9. Ham, Y. S.; Choe, S.; Kim, M. J.; Lim, T.; Kim, S. K.; Kim, J. J., Electrodeposited Ag Catalysts for the Electrochemical Reduction of CO_2 to CO. *Appl. Catal. B-Environ.* **2017,** 208, 35-43.
10. Gao, F. Y.; Hu, S. J.; Zhang, X. L.; Zheng, Y. R.; Wang, H. J.; Niu, Z. Z.; Yang, P. P.; Bao, R. C.; Ma, T.; Dang, Z.; Guan, Y.; Zheng, X. S.; Zheng, X.; Zhu, J. F.; Gao, M. R.; Yu, S. H., High-Curvature Transition-Metal Chalcogenide Nanostructures with a Pronounced Proximity Effect Enable Fast and Selective CO_2 Electroreduction. *Angew. Chem. Int. Edit.* **2020,** 59, (22), 8706-8712.
